# Supplementary material for: An artificial miRNA system reveals that relative contribution of translational inhibition to miRNA-mediated regulation depends on environmental and developmental factors in Arabidopsis thaliana
Source: PLoS One. 2018 Feb 16;13(2):e0192984. doi: 10.1371/journal.pone.0192984 (PMC5815599; doi:10.1371/journal.pone.0192984)
Supplement: S1 Table — (DOCX) [file pone.0192984.s001.docx]

| Gene | Primer sequence 5´-3´ | Purpose |
| --- | --- | --- |
| DCL1 | TCAAGTAGAAGAACCGCAGCTG | qRT-PCR |
|  | AAGAGATTTACGTTTGGGGTAAGAG | qRT-PCR |
| HYL1 | TCCACTGATGTTTCCTCTGG | qRT-PCR |
|  | GATCTCATAAACAGGCGTTGG | qRT-PCR |
| DRB2 | CCAGTCTTGCCTGATAGTCTTG | qRT-PCR |
|  | CGGTTCTCCTTTCGATGAAT | qRT-PCR |
| SERRATE | CACAGAAGGTGGCAAAGGAT | qRT-PCR |
|  | CGACAAGCTCCTGTAATCAATAAC | qRT-PCR |
| CPL1 | GATCAATGCTTGGTCAACCTCTGC | qRT-PCR |
|  | TTAAGAGTATCTTCCCGAAGATGGC | qRT-PCR |
| AGO1 | TCAGCAGTAGAACATGACACG | qRT-PCR |
|  | TCGGTGGACAGAAGTGGGAATA | qRT-PCR |
| AGO10 | GAATTCGACTTCTACCTTTGTAGCCATGCG | qRT-PCR |
|  | CGCTGGAGGAACTATAGAGACCGAC | qRT-PCR |
| LUCIFERASE | GCTCACTGAGACTACATCAGC | qRT-PCR |
|  | CGCTTCCGGATTGTTTACAT | qRT-PCR |
| TUBULINE | GAGCCTTACAACGCTACTCTGTCTGTC | qRT-PCR |
|  | ACACCAGACATAGTAGCAGAAATCAAG | qRT-PCR |
| miR156 | GCGGCGGTGACAGAAGAGAGT | qRT-PCR |
|  | GTCGTATCCAGTGCAGGGTCCGAGGTATTCGCACTGGATACGACGTGCTC | cDNA synthesis |
| AmiR-LUC | GGCGGCAGTTAACGCCCAGCGTT | qRT-PCR |
|  | GTCGTATCCAGTGCAGGGTCCGAGGTATTCGCACTGGATACGACCGGGAA | cDNA synthesis |
|  | CGGGAAAACGCTGGGCGTTAA | Blot probe |
| U6 | GCTAATCTTCTCTGTATCGTTCC | Blot probe |
| Universal | GTGCAGGGTCCGAGGT | qRT-PCR |
